# Prediction of Acute Mammalian Toxicity Using QSAR Methods: A Case Study of Sulfur Mustard and Its Breakdown Products

**DOI:** 10.3390/molecules17088982

**Published:** 2012-07-27

**Authors:** Patricia Ruiz, Gino Begluitti, Terry Tincher, John Wheeler, Moiz Mumtaz

**Affiliations:** 1 Computational Toxicology and Methods Development Lab, Division of Toxicology and Human Health Sciences, Agency for Toxic Substances and Disease Registry, Atlanta, GA 30333, USA; 2 Environmental Public Health Readiness Branch, National Center for Environmental Health, Atlanta, GA 30333, USA

**Keywords:** sulfur mustard, QSAR, SAR, mammalian oral, LD_50_, quantitative structure-activity relationship, *in silico* modeling

## Abstract

Predicting toxicity quantitatively, using Quantitative Structure Activity Relationships (QSAR), has matured over recent years to the point that the predictions can be used to help identify missing comparison values in a substance’s database. In this manuscript we investigate using the lethal dose that kills fifty percent of a test population (the LD_50_) for determining relative toxicity of a number of substances. In general, the smaller the LD_50_ value, the more toxic the chemical, and the larger the LD_50_ value, the lower the toxicity. When systemic toxicity and other specific toxicity data are unavailable for the chemical(s) of interest, during emergency responses, LD_50_ values may be employed to determine the relative toxicity of a series of chemicals. In the present study, a group of chemical warfare agents and their breakdown products have been evaluated using four available rat oral QSAR LD_50_ models*. *The QSAR analysis shows that the breakdown products of Sulfur Mustard (HD) are predicted to be less toxic than the parent compound as well as other known breakdown products that have known toxicities. The QSAR estimated break down products LD_50_ values ranged from 299 mg/kg to 5,764 mg/kg. This evaluation allows for the ranking and toxicity estimation of compounds for which little toxicity information existed; thus leading to better risk decision making in the field.

## 1. Introduction

**Figure 1:**
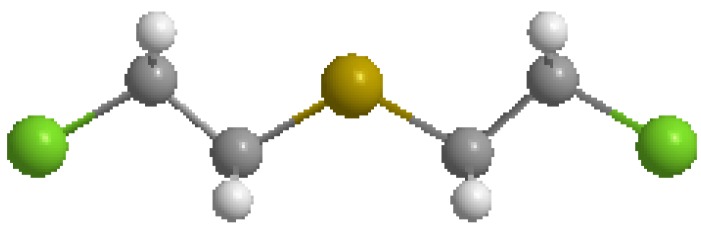
Bis-(2-chloroethyl) sulfide.

From World War I to 1968, the United States produced chemical weapons and warfare agents as a deterrent against use of similar weapons by other countries. Sulfur mustard (HD, Figure 1), a vesicant and alkylating chemical, is one such agent [1,2] Although never used during warfare by the United States, these chemicals are still abundant and are now deteriorating with age. In 1985, the U.S. Congress mandated that the Department of Defense be responsible for establishing a Chemical and Biological Defense (CBD) program, U.S. Code Title 50, Sections 1521 through 153, and provide for chemical weapons disposal and destruction. In accordance with this congressional mandate the Center for Disease Control and Prevention’s (CDC), Environmental Public Health Readiness Branch (EPHRB), Chemical Weapons Elimination section has been tasked with overseeing the Army’s destruction of chemical weapons to ensure that the general population, worker population and environment are protected.

The Pueblo Chemical Agent Pilot Plant (PCAPP) plans to utilize neutralization processes to destroy the HD stored at the Pueblo Chemical Army Depot (PCAD). This process creates several breakdown products during and after the neutralization. The majority of these breakdown products do not have sufficient toxicity data to properly assess the human health impacts related to their exposure or to select appropriate personnel protective equipment (PPE) which will ensure the safety of the personnel. Thus, various stakeholders, viz., state, local, territorial, and tribal public health departments have stated concerns that only are the toxicity databases weak even indicators of overt mammalian toxicity like the lethal dose values that kill fifty percent of test population (LD_50_) are not available for such chemicals. The LD_50_ value is the lethal dose of a substance that will kill 50% of the test animals/organisms within 24 hours of exposure to a chemical [3,4]. LD_50_ values have been used to express the relative hazards associated with the acute toxicity of chemicals and are often used for the initial evaluation of toxicity. Computer-assisted Structure Activity Relationship (SAR) and Quantitative Structure Activity Relationship (QSAR) models, are being increasingly used to fill the data gaps in chemical (pharmaceutical, agrochemical, food additives and other industrial) toxicity databases. These *in silico *tools thus provide a means of assessing toxicity of chemicals that lack appropriate experimental test data [5,6,7,8,9,10,11,12,13].

Most *in silico* prediction systems used to estimate qualitative and quantitative toxicity are either SAR or QSAR models [14,15,16,17,18]. An SAR model, and/or an expert system, establishes qualitative association between a chemical’s substructures and its potential toxicity [19,20]. The confidence in the predictions of a new chemical is based on whether such identified structural alerts are present in the structure of a chemical of interest. Because expert systems are based on qualitative SARs, they usually do not make a quantitative prediction of toxic effect; predictions are expressed in a binary fashion *i.e.*, toxic, indeterminate, or non-toxic. A QSAR model, on the other hand, is a mathematical relationship between the chemical’s quantitative molecular descriptors and its toxicological, biological, and physicochemical activities [9,13,21,22,23]. Molecular descriptors derived from atomic or molecular properties that encode physicochemical, topological, and surface properties of molecules are typically used as the backbone of a predictive QSAR model [21,24,25]. These descriptors are then correlated with a toxicological response of interest through a suitable statistical approach such as linear multiple regression, nearest neighbor, clustering, random forest, discriminant analysis, recursive partitioning, artificial neural networks, *etc. *[26,27,28,29,30,31,32,33,34,35]. Unlike an expert system, a QSAR model provides discrete quantitative predicted values given just the values of the molecular descriptors finally selected in the QSAR model. Choosing the appropriate SAR or QSAR model(s) is a challenge for federal agencies because several such models are available that predict a wide array of endpoints including the LD_50_ values. Additionally, acute oral mammalian toxicity (LD_50_) is one of the more complex toxicological endpoints to predict using QSAR methods. Presence of a gamut of biological and molecular events that lead to variable biological mechanisms and a lack of accurate and true understanding of the mechanism of toxicity contribute to this complexity [8,36].

## 2. Results

A total of 22 chemicals including the parent chemical HD, and its potential breakdown products, experimentally tested or untested, were evaluated using TOPKAT [37], ADMET Predictor [38], and T.E.S.T. [39] (Table 1). Experimental rat oral LD_50_ data for this study was available from two sources, ChemIDplus and Registry of Toxic Effects of Chemical Substances databases.

**Table 1:** Chemical name, structures and experimental LD_50_ of HD and its breakdown products.

Chemical Name	Chemical Structure	Experimental LD_50_ (mg/kg)
Bis (2-chloroethyl) sulfide (HD)	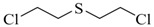	17
Bis [2-(2-chloroethylthioethyl) ether] (T)	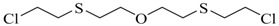	na
1,2-bis(2-chloroethylthio)ethane (Q)	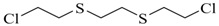	na
Bis (2-chloroethyl) disulfide	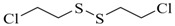	na
Thiodiglycol (TDG)	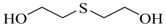	6,610
Thiodiglycol sulfoxide	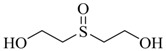	na
1,4-Dithiane	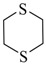	2,768
1,4-Oxathiane	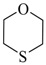	2,830
Bis-[2-(2-hydroxyethylthioethyl)]ether (TOH)	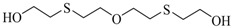	na
Ethanol, 2,2'-[1,2-ethanediylbis(thio)]-bis- (QOH)	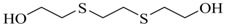	na
1-(2-hydroxyethylthio)-2-(2-vinylthioethoxy)ethane	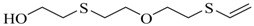	na
1-(2-hydroxyethylthio)-2-(2-vinylthio)ethane	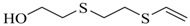	na
2-Hydroxyethyl vinyl sulfide	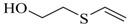	na
2-Methylnaphthalene	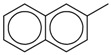	1,630
Bis-(2-chloroethyl) ether	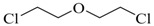	75
Bis-(2-ethylexyl)phthalate	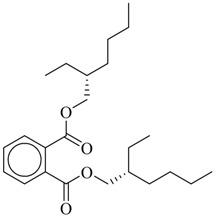	30,600
Propanal	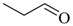	1,410
Ethylene Glycol	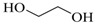	4,700
Ethylene dichloride	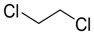	670
Bis-(2-Chloroethyl) sulfoxide	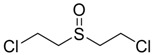	na
Bis-(2-Chloroethyl) sulfone	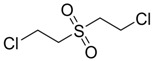	na
Thiodiglycol sulfone	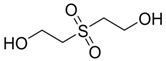	na

na: not available.

### 2.1. TOPKAT Predictions of LD_50_ Values

Of the 22 chemicals queried, eight chemicals [thiodiglycol (TDG), 1,4-oxathiane, 2-methylnaphthalene, bis-(2-chloroethyl) ether, bis-(2-ethylhexyl)phthalate, propanal, ethylene glycol, and ethylene dichloride] were found in the database associated with the TOPKAT LD_50_ model. Seven of the 22 chemicals were outside OPS of the model. Three of these seven were deemed acceptable estimates because the chemicals were outside the OPS in only one dimension. The remaining four were unacceptable estimates because they were outside the OPS in at least two dimensions.

**Figure 2:**
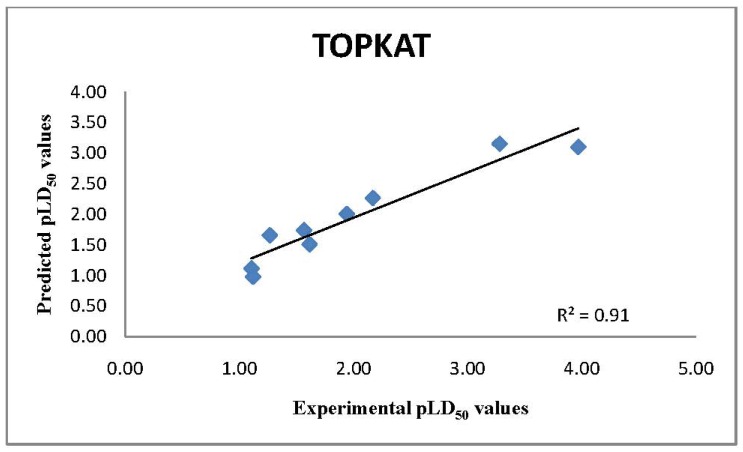
Comparison of Experimental pLD_50_ values with TOPKAT QSAR pLD_50_ model predictions.

The 15 remaining chemicals were very well represented in the LD_50_ model database as assessed by the univariate analysis. The multivariate analysis also showed that these chemicals were within the OPS of the model. The similarity analysis showed that there are several chemicals in the database that have a very close similarity distance. Hence, the confidence in the assessment of LD_50_ values for these chemicals is high. The estimated LD_50_ value of the break down products were within a factor of 4 (Figure 2) of the experimental values as shown in Table 2. This is well within a default factor of 10 often used in traditional risk assessment of environmental chemicals to compensate for uncertainties [40]. 

**Table 2:** Comparison of the estimated LD_50_ values using TOPKAT, 2D and 3D ADMET predictor, and the T.E.S.T model for chemicals with available experimental data.

Chemical Name	Experimental LD_50_*	TOPKAT	ADMET 2D	ADMET 3D	T.E.S.T Consensus
Bis (2-chloroethyl) sulfide	17	125.9	536.84	349.32	38.69
Thiodiglycol (TDG)	6,608.56	2,700	1,651.93	1,407.24	5,426.53
1,4-Dithiane	2,767.71	OPS	502.47	762.79	1,602.59
1,4-Oxathiane	2,830.26	1,900	791.98	1,185.75	1,834.75
2-Methylnaphthalene	1,629.3	1,400	1,884.9	1,864.48	1,391.74
Bis-(2-chloroethyl) ether	75	100.8	215.35	130.57	44.72
Bis-(2-ethylhexyl)phthalate	30,600	>10,000	8,781.91	6,791.82	37,293.36
Propanal	1,409.62	1,800	404.01	427.17	353.02
Ethylene glycol	4,700	6,500	2,705.28	2,034.17	2,587.11
Ethylene dichloride	670	537.5	298.37	297.55	811.49

LD_50_* = mg/kg unit; OPS: outside of optimum prediction space.

### 2.2. ADMET Predictor LD_50_ Values

**Figure 3:**
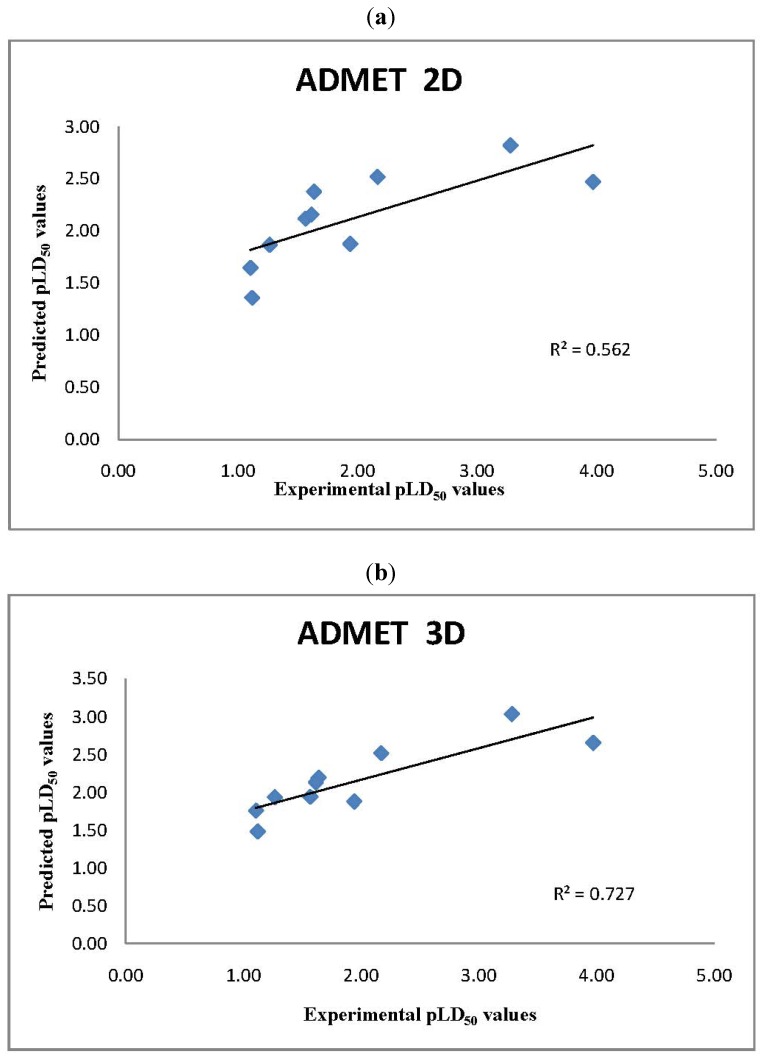
(**a**) Comparison of Experimental pLD50 values with 2D ADMET predictor QSAR pLD_50_ model predictions; (**b**) Comparison of Experimental pLD50 values with 3D ADMET predictor QSAR pLD_50_ model predictions.

Of the 22 chemicals queried, 10 chemicals [bis(2-chloroethyl) sulfide, thiodiglycol, 1,4-dithiane, 1,4-oxathiane, 2-methylnaphthalene, bis-(2-chloroethyl) ether, bis-(2-ethylhexyl)phthalate, propanal, ethylene glycol and ethylene dichloride) were found in the database associated with the ADMET 2D and 3D *Predictor *LD_50_ model as shown in Table 2. All the chemicals were within the applicability domain of the models, and met the requirements of constraints, hence the confidence in all the predicted LD_50_ values is high Figure 3a,b. The predicted LD_50_ values of HD and its breakdown products were within a factor of five, much smaller than a default factor of 10 often used in chemical risk assessments to compensate for uncertainties (Table 2).

### 2.3. T.E.S.T. Predictions of LD_50_ Values

**Figure 4:**
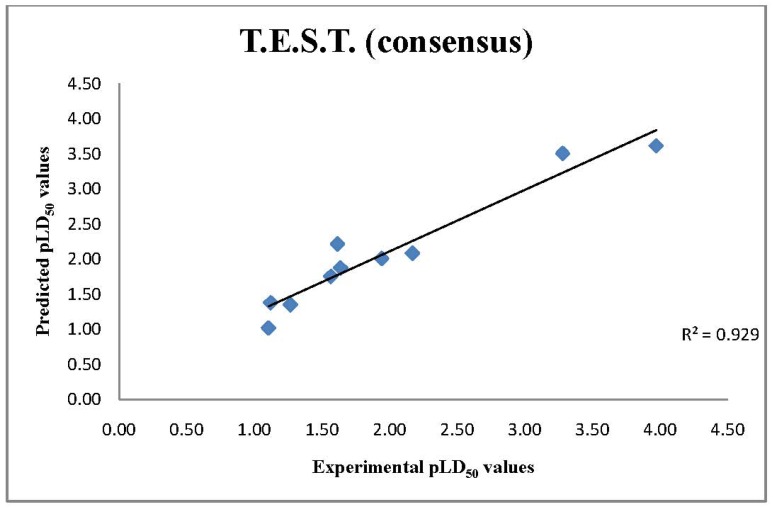
Comparison of Experimental pLD50 values with T.E.S.T QSAR pLD_50_ consensus model predictions.

Of the 22 chemicals queried, seven chemicals [bis(2-chloroethyl) sulfide, 1,4-oxathiane, 2-methyl-naphthalene, bis-(2-chloroethyl) ether, ethylene glycol, propanal and ethylene dichloride) were found in the model database associated with the consensus T.E.S.T LD_50_ model, and two (1,4-dithiane and thiodiglycol) were used in the external test set. All 22 chemicals were inside the applicability domain of the model. The 13 remaining chemicals were very well represented in the model database as assessed by the statistical analysis. The similarity analysis showed that there are several chemicals in the database that have very close similarity distance. Hence, the confidence in the assessment of LD50 values for these chemicals is high (Figure 4).

The estimated LD_50_ value of HD and its breakdown products were within a factor of less than two (Table 2). This is well within a default factor of 10 often used in risk assessment of environmental chemicals to compensate for uncertainties. The estimated LD_50_ values for the breakdown products that lack experimental data are shown on Table 3. Six chemicals [1,2-bis(2-chloroethylthio)ethane, bis-[2-(2-hydroxyethylthioethyl)]ether, ethanol, 2,2'-[1,2-ethanediylbis(thio)]-bis-, 1-(2-hydroxyethylthio)-2-(2-vinylthioethoxy)ethane, 1-(2-hydroxyethylthio)-2-(2-vinylthio)ethane, 2-hydroxyethyl vinyl sulfide) were found outside of the optimum prediction space for the TOPKAT model. However, ADMET Predictor and T.E.S.T. (consensus) LD_50_ model could estimate the LD_50_ values of these chemicals.

**Table 3:** Comparison of the estimated LD_50_ values using TOPKAT, 2D, 3D ADMET predictor and the T.E.S.T. model for chemicals that lack experimental data.

Chemical Name	TOPKAT	ADMET 2D	ADMET 3D	T.E.S.T (consensus)
Bis [2-(2-chloroethylthioethyl) ether] (T)	816.1	197.18	110.7	166.96
1,2-Bis(2-chloroethylthio)ethane (Q)	OPS	630.39	369.39	93.02
Bis (2-chloroethyl) disulfide	153.6	512.3	443.37	351.90
Thiodiglycol sulfoxide	1,400	3,524.73	2,912.39	4,094.75
Bis-[2-(2-hydroxyethylthioethyl)]ether (TOH)	OPS	1,355.55	1,665.45	14,272.34
Ethanol, 2,2'-[1,2-ethanediylbis(thio)]-bis- (QOH)	OPS	1,014.26	1,045.92	3,506.17
1-(2-Hydroxyethylthio)-2-(2-vinylthioethoxy)-ethane	OPS	574.96	649.36	2,730.21
1-(2-Hydroxyethylthio)-2-(2-vinylthio)ethane	OPS	391.58	399	1,339.93
2-Hydroxyethyl vinyl sulfide	OPS	474.79	448.1	979.11
Bis-(2-chloroethyl) sulfoxide	67.9	449.01	265.64	577.05
Bis-(2-chloroethyl) sulfone	227	462.07	260.21	249.89
Thiodiglycol sulfone	2,800	2,715.91	1,805.24	12,190.02

OPS: outside of l optimum prediction space.

## 3. Discussion

Sulfur mustard neutralization breakdown products are a diverse group of chemicals that either have no or minimal test data available regarding their toxicological effects [1]. Some members of this group and their breakdown products even lack LD_50_ values. Hence a 2-step *in silico* approach was adopted wherein a QSAR analysis was performed. In the first step we estimated LD_50_ values using four different QSAR LD_50_ models. Each one of the models is internally and externally validated, robust, and has good applicability domains. The LD_50_ predicted values were compared with the available experimental values when available, and were found to be within acceptable ranges (10 fold) used in chemical risk assessment. Analyses were performed to evaluate the use of these chemicals in the training set used for the model development, if they were part of the external validation set, or they were not used in the model development. Through this process we could evaluate which of the chemicals in this assessment were used to develop each of the models, thus we can identify the applicability domain of each model.

In the second step, using the above models, we determined the LD_50_ values for breakdown products that lack experimental data (Table 3). The key application of QSAR is to fill the data gaps for chemical toxicity. We achieved this through considerations of the strengths and weaknesses of each model, knowing that none of them is a perfect model. If the strength and advantage of a model is clearly known, the output of such a model can be used easily. TOPKAT model is well known and used by regulatory community, particularly federal agencies. The applicability domain of the model for this particular data set of chemicals was poor, *i.e.*, seven of the twenty two chemical were outside the optimum prediction space (OPS). On the contrary, ADMET predictor and T.E.S.T. had high chemical applicability domain for this data set. These models use both internal and external validated compared with TOPKAT that uses only one validation method. These models not only showed a high chemical applicability domain but also broad activity ranges in this applicability domain for this data set. However, the T.E.S.T model had superior performance compared to ADMET predictor.

Taking an average of estimates when using multiple models could compensate the limitations of the individual models that use different descriptors and statistical methods to model different aspects of the toxicological affects. Thus, the use of a consensus model is always more beneficial than an individual.

When using multiple models with varying modeling techniques (molecular descriptors, statistical methods and validation), it is a bigger challenge to judge the model output if their performances are comparable but slightly different [30,41,42,43,44]. In such cases, the conventional wisdom is to use an arithmetic averaging scheme which was also used in our study (Table 4), or use the most conservative values estimated by the models. For example, as shown in Table 3, the estimated LD_50_ values of bis-[2-(2-hydroxyethylthioethyl)] ether (TOH) and thiodiglycol sulfone are less toxic by the T.E.S.T. model (much higher LD_50_ values) compared to the other models, in such case, the T.ES.T model estimate could be excluded using a more safe approach.

**Table 4:** Average estimated LD_50_ values and the classification of HD breakdown products that lack of experimental LD_50_ values.

Chemical Name	QSAR estimated LD_50 _(average of TOPKAT, 2D, 3D ADMET predictor and T.E.S.T.)	Toxicity Class *
Bis [2-(2-chloroethylthioethyl) ether] (T)	322.74	Very Toxic
1,2-Bis(2-chloroethylthio)ethane (Q)	364.27	Very Toxic
Bis (2-chloroethyl) disulfide	365.29	Very Toxic
Thiodiglycol sulfoxide	2,982.97	Moderately Toxic
Bis-[2-(2-hydroxyethylthioethyl)]ether (TOH)	5,764.45	Slightly Toxic
Ethanol, 2,2'-[1,2-ethanediylbis(thio)]-bis- (QOH)	1,855.45	Moderately Toxic
1-(2-hydroxyethylthio)-2-(2-vinylthioethoxy)-ethane	1,318.18	Moderately Toxic
1-(2-Hydroxyethylthio)-2-(2-vinylthio)ethane	710.17	Moderately Toxic
2-Hydroxyethyl vinyl sulfide	634.00	Moderately Toxic
Bis-(2-Chloroethyl) sulfoxide	339.90	Very Toxic
Bis-(2-Chloroethyl) sulfone	299.79	Very Toxic
Thiodiglycol sulfone	4,877.79	Moderately Toxic

* Super toxic (<5 mg/kg), extremely toxic (5–50 mg/kg), very toxic (50–500 mg/kg), moderately toxic (500–5,000 mg/kg), slightly toxic (5,000–15,000 mg/kg) and practically non-toxic (>15,000 mg/kg).

With an increase in the number of QSAR models available, commercially or open source, that are developed using a variety of approaches, the final interpretation and application will depend on the user confidence and the transparency of the model [44,45,46,47] The LD_50_ values, experimental or QSAR, are used to determine the relative toxicity of a series of chemicals in which the LD_50_ value of a given chemical is compared with the LD_50_ values of other chemicals [4]. Using acceptable toxicity scales, the chemicals are assigned to various groups. One of the most common scales used is the Gosselin, Smith and Hodge scale. Super toxic (<5 mg/kg), extremely toxic (5–50 mg/kg), very toxic (50–500 mg/kg), moderately toxic (500–5,000 mg/kg), slightly toxic (5,000–15,000 mg/kg) and practically non-toxic (>15,000 mg/kg) [48]. 

**Scheme 1:**
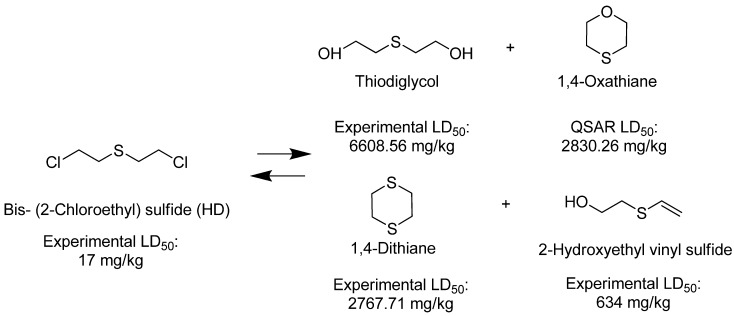
Hydrolysis of bis-(2-chloroethyl) sulfide.

Using this scale, the QSAR estimates enabled the ranking of these chemicals on the basis of potential toxicities, in a rapid manner. The breakdown products of sulfur mustard (HD) hydrolysis reaction, thiodiglycol (TDG), 1,4-dithiane, 1,4-oxathiane and 2-hydroxyethyl vinyl sulfide (Scheme 1) were estimated to have moderate LD_50_ values (>500 mg/kg), when compared with HD, the parent compound.

**Scheme 2:**
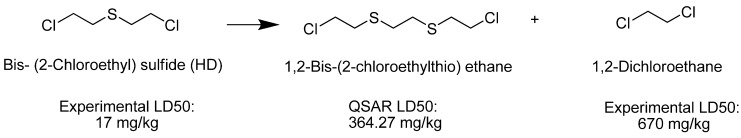
Dissolution of Bis-(2-Chloroethyl) sulfide.

The dissolution of HD in presence of water leads to the formation of the intermediate sulfonium ion which in turn reacts with another molecule of HD to form 1,2-bis-(2-chloroethylthio) ethane (Q) and 1,2-dichloroethane (Scheme 2). The estimated LD_50_ values of 1, 2-bis-(2-chloroethylthio) ethane is <500 mg/kg which is very toxic, but less than HD.

**Scheme 3:**
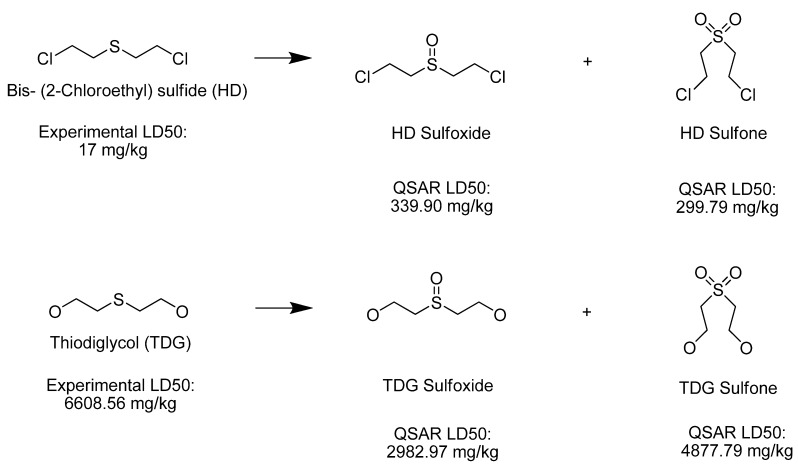
Oxidation of Bis-(2-Chloroethyl) sulfide.

HD and its hydrolysis product TDG are oxidized to give the sulfoxide and sulfone analogs of HD and TDG (Scheme 3). The sulfoxide and sulfone analogs of HD are less toxic than HD; the estimated LD_50_ values were between 339.90 and 299.79 mg/kg, respectively. TDG and its sulfoxide and sulfone analogs are slightly and moderately toxic, respectively, when compared to the extremely toxic parent compound, HD.

**Figure 5:**
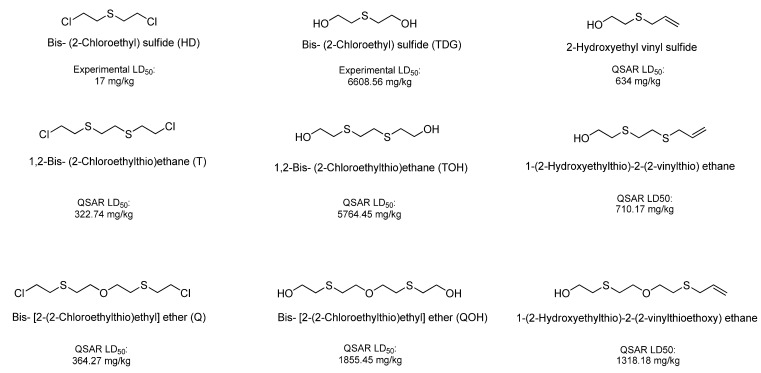
Sulfur mustard (HD), O-mustard (T) and sesquimustard (Q), and their hydroxylated and vinyl analogs.

The estimated LD_50_ values of HD, O-mustard (T) and sesquimustard (Q) and their hydroxylated analogs TDG, TOH and QOH show that these analogs are much less toxic (Figure 8). However, when compared with their vinyl analogs, TDG, TOH and QOH are shown to have a higher potential toxicity, but less than HD, T and Q (Figure 5).

The QSAR estimated LD_50_ values for the breakdown products ranged from 299.79 mg/kg to 5,764.45 mg/kg. These data indicate that five breakdown products fall under very toxic class (50–500 mg/kg) while seven fall under moderately toxic (LD_50_ values 0.5–5 g/kg) class. None of the chemicals fall under the extremely toxic or super toxic categories (Table 4). Thus, the experimentally untested breakdown products are potentially less toxic than those chemicals that were experimentally tested (range from 17 mg/kg to 30,600 mg/kg). The experimentally tested chemicals including the parent chemical fall under the extremely toxic class (5–50 mg/kg) and a breakdown product falls under the very toxic (50–500 mg/kg) class. Six of the breakdown products fall under moderately toxic (LD_50_ values 500–5,000 mg/kg), while one each falls under slightly toxic (5,000–15,000 mg/kg) and practically non toxic (>15,000 mg/kg) class. None fall under super toxic class (less than 5 mg/Kg). Thus, overall the potential toxicity of QSAR estimated breakdown products is potentially lower than the parent compound. The QSAR LD_50_ models estimates not only are within a factor of 10 of the experimental available data [except for bis(2-chloroethyl) sulfide], but also have shown an overall degree of conservatism, except for the HD, which was predicted as very toxic by TOPKAT, ADMET 2D and 3D models, compared to extremely toxic experimental value. This information will be useful to stakeholders involved in HD neutralization processes to assess risk associated with these breakdown products. 

Mammalian toxicity, particularly LD_50_ values are much more challenging because the mechanism of acute lethal toxicity is complex, and is not fully understood because of complex interactions between the organism and pharmacokinetic and pharmaco-dynamics of the chemicals. However, a growing number of *in silico* tools and QSAR models continue to be developed because of demand for such estimates due to resource limitations and timely needs, apart from ethical reasons [49]. Gaining knowledge from these kinds of activities should help *in vitro* to *in vivo* extrapolations, especially since large *in vitro* data generating efforts are ongoing at the National Academy of Science and the EPA [50,51,52]. Through these efforts, meaningful structural features can be identified that will lead to the development of robust predictive models, and specific selection criteria for use of such models. A thoughtful application and prudent use of such models can help estimate the toxicity of chemicals that lack experimental data, and prioritize chemicals for screening and subsequent toxicity testing while saving cost and time, thus minimizing experimental animal testing while optimizing overall use of resources. Recent laws are pushing the acceptance of these methods and their use by the regulatory and public health communities in the mitigation of potential hazardous exposures that could compromise the quality of human health and environment [53,54,55]. *In silico* tools can play a pivotal role for assessing the health risk of environmental and pharmaceutical chemicals, especially when the only characteristic known is the structure of a chemical.

## 4. Materials and Methods

The chemical and biological defense program of the Department of Defense uses multiple chemical reactions such as dissolution, hydrolysis, oxidation, and neutralization (see discussion section) to dispose, decontaminate and destroy chemicals weapons such as sulfur mustard (HD) that have been stored for a long time with varies type of stabilizers.

For the present work, we used the following three QSAR modeling packages to estimate the LD_50_ values of HD [bis-(2-chloroethyl) sulfide (Figure 1)] and its breakdown products: TOPKAT^®^ (Toxicity Prediction by Komputer Assisted Technology, Version 6.2, Accelrys, Inc.) and ADMET Predictor 5.0 (Simulation Plus Inc.) and Toxicity Estimation Software Tool (T.E.S.T. US EPA) [37,38,39].

### 4.1. Toxicity Prediction by Komputer Assisted Technology (TOPKAT)

QSAR software package TOPKAT^®^ 6.2, is a tool for structure-based activity assessment, which correlates activity with structural attributes (descriptors). The software encompasses a set of QSAR models, each concerning a different type of activity. TOPKAT QSAR models have been used for the estimation of potential toxicity such as carcinogenicity, mutagenicity, developmental toxicity, LD_50_, LOAELs and skin sensitization. The TOPKAT QSARs models utilize a computer-based method to assess the activity of a chemical solely based on its molecular attributes [37].

#### TOPKAT Rat Oral LD_50_ Model

The LD_50_ QSAR model of the TOPKAT package comprises 19 statistically significant and cross-validated QSAR sub-models, and the data from which these models are derived. These models are derived from experimental LD_50_ values of approximately 4,000 chemicals from open literature. Each QSAR model assesses the rat oral acute median lethal dose (LD_50_) for a specific class of chemicals. Molecular structure is the only input required to conduct an LD_50_ assessment. This model automatically determines whether the submitted structure belongs in the Optimum Prediction Space (OPS) of the model, and (ii) computes QSAR similarity distance from chemicals with experimental LD_50_ data in order to evaluate the reliability of the QSAR-based assessment [56,57].

### 4.2. ADMET Predictor Rat Oral LD_50_ Model

ADMET Predictor™ is a state-of-the-art computer program designed to estimate certain ADMET (Absorption, Distribution, Metabolism, Elimination, and Toxicity) properties of a chemical from its 2 and 3 dimensional (2D and 3D) molecular structures (Simulations Plus Inc.). The program uses molecular descriptor values as inputs to independent mathematical models (generally, nonlinear machine learning techniques) in order to generate estimates for each of the ADMET properties. Qualitative and quantitative models of several measures of toxicity are estimated by ADMET Predictor including maximum recommended therapeutic dose, fathead minnow lethal toxicity, *Daphnia magna* lethal toxicity, acute rat toxicity, Ames mutagenicity in *Salmonella typhimurium,* carcinogenicity in rats, *etc. *The rat oral LD_50_ model is supported by data from two sources, CDC’s Registry of Toxic Effects of Chemical Substances (RTECS), and the ChemIDplus database. The LD_50_ data was converted to the negative logarithm of LD_50_ (p*LD_50_*) for the model development, 7150 unique identifiable compounds were selected and used. Two models are available that are based on 2 and 3 dimensional (2D and 3D) descriptors of the chemical structure. Greater than (or equal to) 20% of the data were set aside for the external test sets prior to training the models. It is noteworthy that the models had complete coverage and were able to make predictions having root mean square error (RMSE) of approximately 0.63 log units for both 2D and 3D test sets. 

### 4.3. Toxicity Estimation Software Tool (T.E.S.T.)

T.E.S.T. estimates toxicity using a variety of QSAR methodologies, such as hierarchical clustering, the Food and Drug Administration (FDA) MDL, nearest neighbor, and a consensus model which is simply the average of the predicted toxicities from other QSAR methodologies (taking into account the applicability domain of each method) [30]. The required descriptors are calculated without requiring any external programs. The structure of a chemical can be simply entered through the use of multiple tools including a chemical sketcher window, a text file containing SMILES notations, or importing it from a database of structures. After entering the structure, a chemical’s toxicity can be estimated using one of several advanced methodologies. T.E.S.T. version 4.0 contains LD_50_ values from 7,420 chemicals [28].

#### 4.3.1. The Hierarchical Clustering Method

The hierarchical clustering method utilizes a variation of the Ward’s Minimum Variance Clustering Method [58] to produce a series of clusters from the initial training set. For a training set of *n* chemicals, initially there will be *n* clusters formed. At each step in the clustering process, two clusters are combined so that the increase in variance over all of the clusters in the system is minimized. The change in variance caused by combining clusters *j* and *k* is as follows:


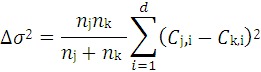
(1)

where *n_j_* = number of chemicals in cluster *j*, *C_j_*_,*i*_ is the centroid (or average value) for descriptor *i* for cluster *j*, and *d* is the number of descriptors (~800) [28]. The process of combining clusters while minimizing variance continues until all of the chemicals are lumped into a single cluster. After the clustering is complete, each cluster is analyzed to determine if an acceptable QSAR model can be developed. A genetic algorithm technique is used to select descriptors to build a multilinear regression model for each cluster [28], and each model must achieve a leave-one-out cross-validation (LOO-CV) accuracy of 0.5 to be used in making predictions. The predicted value for a given test chemical is calculated using the equally weighted average of the model predictions from the closest cluster from each step in the hierarchical clustering. 

#### 4.3.2. The FDA MDL QSAR Method

The FDA MDL method is based on the work of Contrera and coworkers [59]. In this method, predictions for each test chemical are made using a unique cluster (constructed at runtime) which contains structurally similar chemicals selected from the overall training set. This is in contrast to the Hierarchical method, where the predictions are made using one or more clusters that are constructed *a priori* using Ward’s method. For each test chemical, a cluster is constructed using the 30 most similar chemicals from the training set as defined by the cosine similarity coefficient, *SC_i_*_,*k*_, which is calculated as follows: 


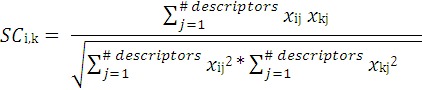
(2)

where *x_ij_* is the value of the *j*-th normalized descriptor for chemical *i* (normalized with respect to all of the chemicals in the original training set) and *x_kj_* is the value of the *j*-th descriptor for chemical *k*. The entire pool of approximately 800 descriptors is used to calculate the similarity coefficient in equation 2. A multiple linear regression model is then built for the new cluster using a genetic algorithm-based method, and the toxicity is predicted [30].

#### 4.3.3. The Nearest Neighbor Method

The nearest neighbor method is a simplification of the variable selection *k*NN approach. In the nearest neighbor method, the toxicity is simply predicted as the average of the toxicity of the three most similar chemicals from the training set. The similarity is defined in terms of the cosine similarity coefficient (Equation 2). In this method, the entire available descriptor pool is used to characterize molecular similarity (as opposed to a subset of the descriptor pool as in the descriptor selection *k*NN method). To make a prediction, each of the neighbors in the training set must exceed a minimum cosine similarity coefficient of 0.5 [28]. 

### 4.4. Applicability Domains (ADs)

Every QSAR model can predict the potential toxicity of any chemical but the confidence in such predictions can vary. However, because each model is developed using a training set of chemicals that cover only a small fraction of the entire chemical universe its prediction capability is restricted to its applicability domain (AD), *i.e.*, its descriptor space. As a consequence of this only a certain fraction of chemicals of an external data set can be reasonably predicted. So it is important to determine if a chemical of interest falls within the AD of a model. If it falls outside the AD, varying degrees of uncertainties could be associated with such a prediction. Model ADs, their characteristics and limitations need to be understood thoroughly for the appropriate interpretation of results [29,31,32,60,61,62,63]. Described below are the AD restrictions or requirements of the models we used in this study.

#### 4.4.1. AD of the TOPKAT Model

In the TOPKAT model two types of analysis, the univariate analysis and multivariate analysis, are automatically performed to determine if a chemical is within the AD of a model. The univariate analysis or coverage examination checks whether all of the structural fragments of the query structure are covered by the chemical database associated with the model. The multivariate analysis or Optimum Prediction Space (OPS) examination checks to see whether the submitted structure fits within or near the periphery of OPS of the equation. If the query compound is deemed outside the OPS a warning about acceptability of the assessment is issued [37]. The details of the assessment process using the TOPKAT software have been published previously [13,37,56,57]. Another feature of TOPKAT, the “QSAR similarity” analysis, enhances the confidence in a prediction [13,57,64]. To assign high, moderate, or low confidence in a prediction the nearest 4 neighbors in the data base with a similarity distance of <0.25 are considered [13].

#### 4.4.2. AD of the ADMET Predictor Model

The ADMET Predictor automatically determines whether a given compound is within the AD of the model. The AD is defined in terms of molecular descriptor space, not in terms of the relative value of an ADMET property. Let *X* denote an ADMET model with known training set and let 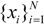 be a descriptor set of model *X*. For each descriptor, *x_i_*(*i*= 1,…, *N*), its minimum and maximum values, 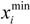 and 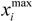, are determined over the training set of *X*. A new compound C is said to be within the scope of *X* if the value of each relevant descriptor *c_i_*(*i*= 1,…, *N*) calculated for C is contained within the corresponding interval 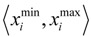 with tolerance equal to 10% of the interval length. Such a compound has its *X* value typed in black bold font. Otherwise, the compound in question is outside the scope of *X* and its *X* value is marked by magenta font (Simulations Plus Inc.).

#### 4.4.3. AD of the Hierarchical Clustering Method

The first restriction, the model ellipsoid constraint, checks if the test chemical is within the multidimensional ellipsoid defined by the ranges of descriptor values for the chemicals in its database. It is satisfied if the leverage of the test chemical (*h*_00_) is less than the maximum leverage value for all of the chemicals used in the model. The second restriction, the *R*_max_ constraint, checks if the distance from the test chemical to the centroid of the database is less than the maximum distance for any chemical in the database to the database centroid. The final constraint, the fragment constraint, stipulates that the chemicals in the database must contain at least one example of each of the fragments that are present in the test chemical [28,30].

#### 4.4.4. AD of the FDA MDL QSAR Method

For the prediction to be valid, three restrictions must be met. The first two, the model ellipsoid and fragment, are same as described above. Third restriction is that the predicted toxicity value must be within the range of experimental toxicity values for the chemicals in the database used to build the model [28,30].

#### 4.4.5. AD of the Nearest Neighbor Method

Predictions using this model require at least three chemicals in the training set that are sufficiently similar to the test chemical. That is, the similarity coefficient between each of the three chemicals and the test chemical in equation 1 must exceed 0.5 [30].

## 5. Conclusions

This study has integrated commercial and open source robust QSAR LD_50_ models to develop an approach for evaluation of the toxicity of chemicals that lack experimental toxicity data. This project was undertaken to determine the potential toxicity of sulfur mustard neutralization breakdown products toxicity using QSAR analysis since there is no experimental toxicity data available for such breakdown products. The QSAR estimates obtained show that the breakdown products will have less potential toxicity than some of the better known breakdown products of sulfur mustard. This evaluation can provide stakeholders with the potential toxicity values to make risk based decisions related to these breakdown products. 
